# Supplementary material for: Discovery of protein-based natural hydrogel from the girdle of the ‘sea cockroach’ Chiton articulatus (Chitonida: Chitonidae)
Source: PeerJ. 2022 May 9;10:e13386. doi: 10.7717/peerj.13386 (PMC9097651; doi:10.7717/peerj.13386)
Supplement: Supplemental Information 3 [file peerj-10-13386-s003.docx]

Supporting Information

Discovery of Protein-Based Natural Hydrogel from the Girdle of the ‘Sea Cockroach’ (*Chiton articulatus*; Chitonidae)

*Emel Çakmak^1,2^, Behlul Koc-Bilican^2,3^, Omar Hernando Avila-Poveda^4,5,6^, Tuğçe Karaduman^2,3^, Demet Cansaran-Duman^7^, Suzanne T. Williams^8^, Murat Kaya^2,3,*^*

^1^ Aksaray University, Güzelyurt Vocational School, Department of Vegetable and Animal

Production, Güzelyurt, Aksaray, Turkey

^2^ASUBTAM- Aksaray University, Science and Technology Application and Research Center, 68100 Aksaray, Turkey

^3^ Department of Molecular Biology and Genetic, Faculty of Science and Letters, Aksaray University, Aksaray, Turkey

^4^ Facultad de Ciencias del Mar, Universidad Autónoma de Sinaloa. Mazatlán, Sinaloa, México

^5^ Programa Investigadoras e Investigadores por México, Consejo Nacional de Ciencia y Tecnología. Ciudad de México, México

^6^ Project Quitón del Pacífico tropical mexicano. Mazatlán, Sinaloa, México

^7^Ankara University, Biotechnology Institute, Kecioren, 06350, Ankara, Turkey

^8^ Natural History Museum, Department of Life Sciences, Cromwell Road, London SW7 5BD, UK

*Corresponding author: Tel.: (+90) 3822882216, E-mail: [muratkaya3806@gmail.com](mailto:muratkaya3806@gmail.com)

**Contents**

**Figure S1**. The process of acquiring the girdle from the *Chiton articulatus* (Mollusca: Polyplacophora). (A) A fresh specimen showing the naked girdle (hyponotum), gills in mantle cavity and foot, (ventral view) (B) The body scleritome and girdle, (dorsal view), (C) The sun-dried, body scleritome and girdle, (D) The softened girdle structure obtained intact, (*) Close-up of the naked girdle (hyponotum, on ventral side) and gills in the mantle cavity………… 3

**Table S1.** Peaks recorded during FTIR analysis in this study for *C. articulatus* hydrogel and their chemical assignment, compared with peaks observed in three other studies of natural

hydrogels.……………………………………………………………………………………... 4

**Figures**


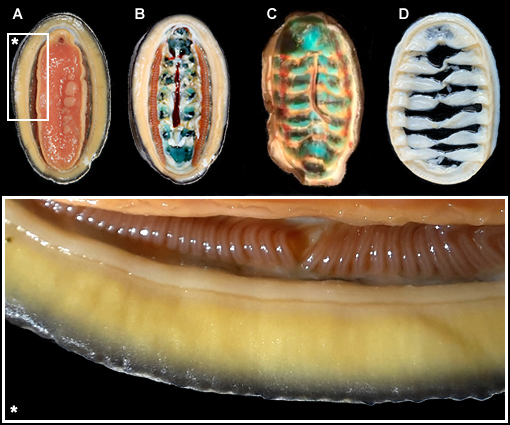


**Figure S1.** The process of acquiring the girdle from the *Chiton articulatus* (Mollusca: Polyplacophora). (A) A fresh specimen showing the naked girdle (hyponotum), gills in mantle cavity and foot, (ventral view) (B) The body scleritome and girdle, (dorsal view), (C) The sun-dried, body scleritome and girdle, (D) The softened girdle structure obtained intact, (*) Close-up of the naked girdle (hyponotum, on ventral side) and gills in the mantle cavity.

**Tables**

**Table S1.** Peaks recorded during FTIR analysis in this study for *C. articulatus* hydrogel and their chemical assignment, compared with peaks observed in three other studies of natural hydrogels.

| **Functional groups and vibration modes** | **Classification** | **Wavenumber (cm^−1^) frequency** | | | |
| --- | --- | --- | --- | --- | --- |
|  |  | **Hydrogel from**  ***C. articulatus*** | **Collagen**  **[93]** | **Keratin**  **[94]** | **Fibroin**  **[95]** |
| N-H stretching |  | 3275 | - | - | 3280 |
| CH3 sym. stretch and CH2asym. stretch | Aliphatic compounds | 2921 | - | - | 2930 |
| C=O secondary amide stretch | Amide I | 1625 | 1628 | 1628 | 1630 |
| N–H bend, C–N stretch | Amide II | 1537 | 1540 | 1531 | 1520 |
| CH2 bending and CH3 deformation | - | 1457 | 1449 | - | 1439 |
| CH bend, CH3 sym. deformation | - | 1404 | 1397 | - | - |
| CH2 wagging | Amide III, components of protein | 1235 | 1234 | 1247 | 1230 |
